# Supplementary material for: Exposing 24-hour cycles in bile acids of male humans
Source: Nat Commun. 2024 Nov 19;15:10014. doi: 10.1038/s41467-024-53673-9 (PMC11576969; doi:10.1038/s41467-024-53673-9)

Exposing 24-hour cycles in bile acids of male humans

Supplementary Information

Extended methodology.

Inclusion/exclusion criteria for the sleep laboratory studies. Eligibility of the participants for the study was determined by completion of validated sleep questionnaires (Pittsburgh Sleep Quality Index (PSQI) $\leq$ 5, Beck Depression Inventory < 10, Epworth Sleepiness Scale < 10, and Horne-Östberg and Munich Chronotype questionnaire), medical and physical assessments, and analysis of blood and urine screening samples. Individuals with extreme chronotypes were excluded. Inclusion criteria included: age between 18 and 35 years; passing a medical assessment; consent to contacting the candidate's general practitioner for confirming the candidate's medical history; agreement to refrain from alcohol, caffeine, exercise, and bright light for 72 h before and during the in-laboratory session; refraining from taking any over-the-counter (including nonsteroidal anti-inflammatory drugs) or prescribed medication for a washout period of 7 days prior to the laboratory session; completion and fulfilment of the defined criteria of the pre-study questionnaires; agreement to eat standardized meals for 48 h prior to the laboratory session; reporting a habitual, regular sleep-wake cycle for the month preceding screening that involved going to bed between 2200 and 0100 h and awaking between 0600 and 0900 h with 6 to 9 h in bed; agreement to keep a regular sleep/wake schedule for the duration of the study; and wearing Actiwatches and completing written sleep diaries for the duration of the study. Participants were excluded for the following: ever having a history of systemic, psychiatric, or neurological disease or drug and alcohol abuse; being a smoker or having been a smoker in the 6 months prior to their screening visit; drinking >21 units of alcohol per week; having a positive drugs of abuse urine screen at screening or upon entry into the laboratory session; having a positive cotinine urine screen at screening or upon entry into the laboratory session; having a body mass index (BMI) <19 or >30 kg/m^2 or a total body weight <50 kg as assessed at the screening visit; being a vegetarian or having other dietary restrictions as this can affect metabolism; taking regular medication that affects melatonin synthesis or circadian rhythms in the past 6 months; having donated over 400 ml of blood within 3 months of screening for the study; having abnormal blood biochemistry and/or hematology as deemed significant by the study physician; being positive for human immunodeficiency virus or hepatitis B or C; having a clinically significant allergy; being considered unsafe to participate as determined by the medical investigator; or having received

any investigational drug and/or participate in any clinical trial within 3 months of the screening assessment.

Calorie intake and macronutrient composition in the entrained protocol. In the entrained protocol, participants were given 2,500 calories per day, divided into 3 x 30% for meals and 1 x 10% for a snack before bedtime. The makeup of macronutrient composition was based on UK dietary guidelines of 50% carbohydrate, 35% fat, and 15% protein (**Supplementary Table S2**). Breakfast was provided at 07:00 h and consisted of cornflakes, milk, wholemeal toast, butter, jam, orange squash (standard portions). Lunch was provided at 13:00 h and consisted of cheese sandwich, yogurt, piece of fruit, orange squash (standard portions). Dinner was provided at 19:00 h and included lasagne, yogurt, piece of fruit, orange squash (standard portions). The snack was a Frij milkshake and two digestive biscuits (standard portions) and was provided at 22:00 h. Water was provided *ad libitum*.

Supplementary Figure S1: Individual fits of circulating bile acids following daily cycles. Linear mixed-effect cosinor models for each individual bile acid showing overall and individual fits (n = 15).

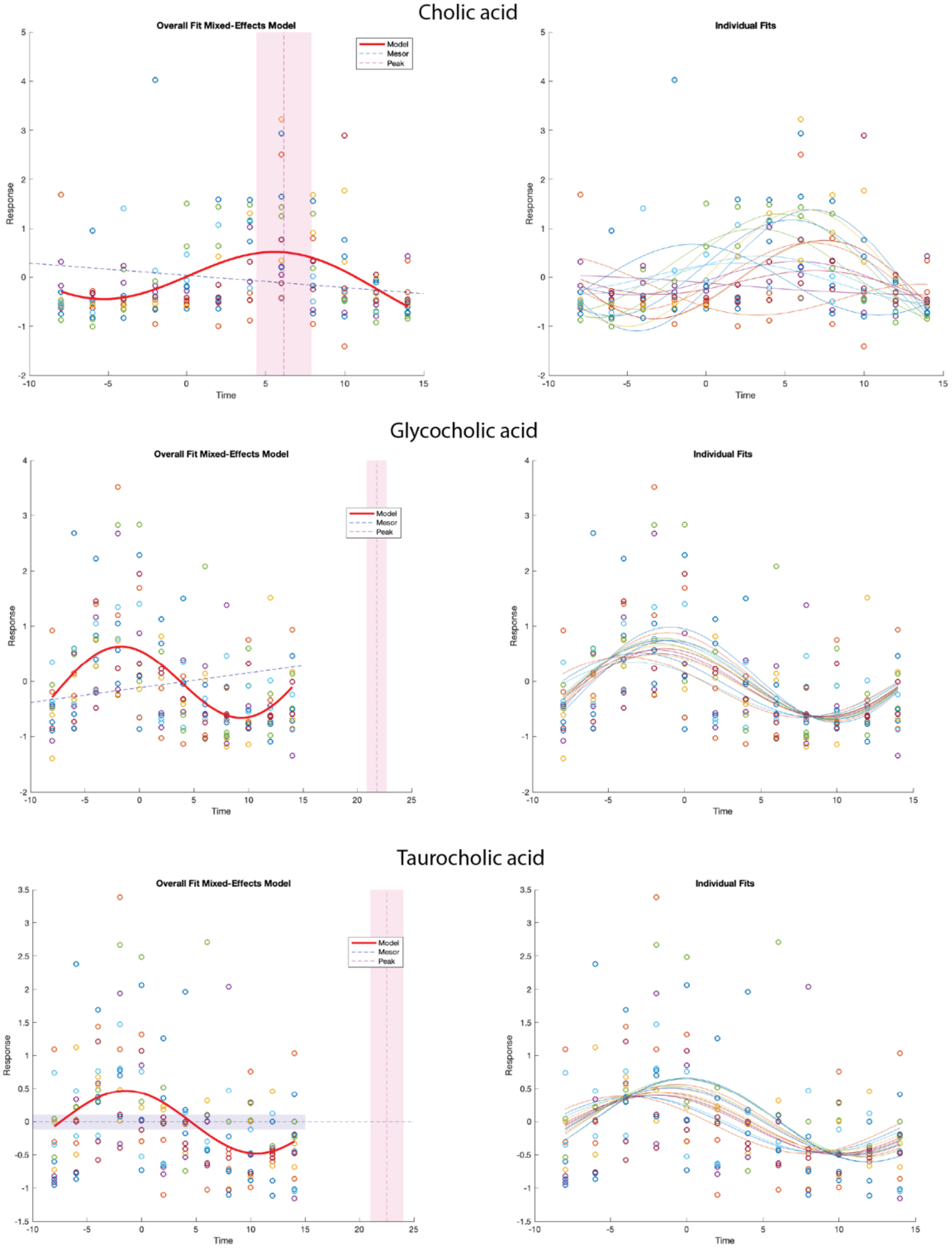

Chenodeoxycholic acid

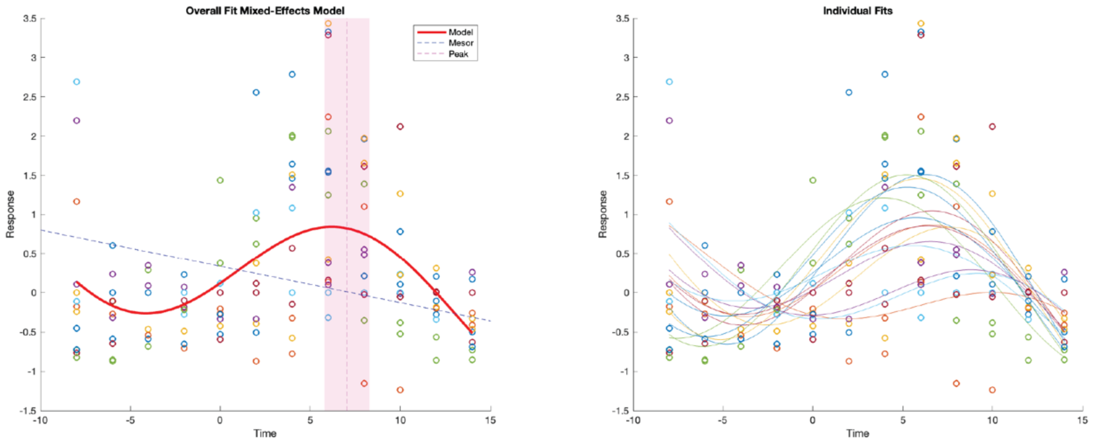

Glycochenodeoxycholic acid

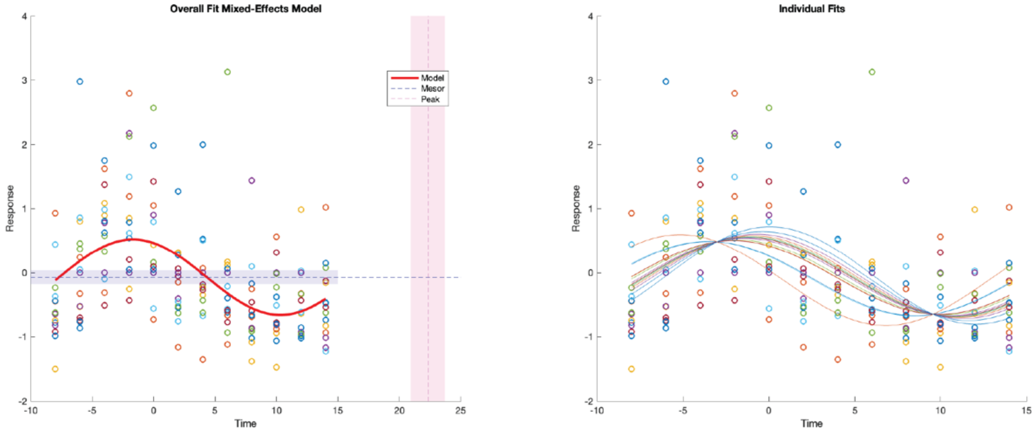

Taurochenodeoxycholic acid

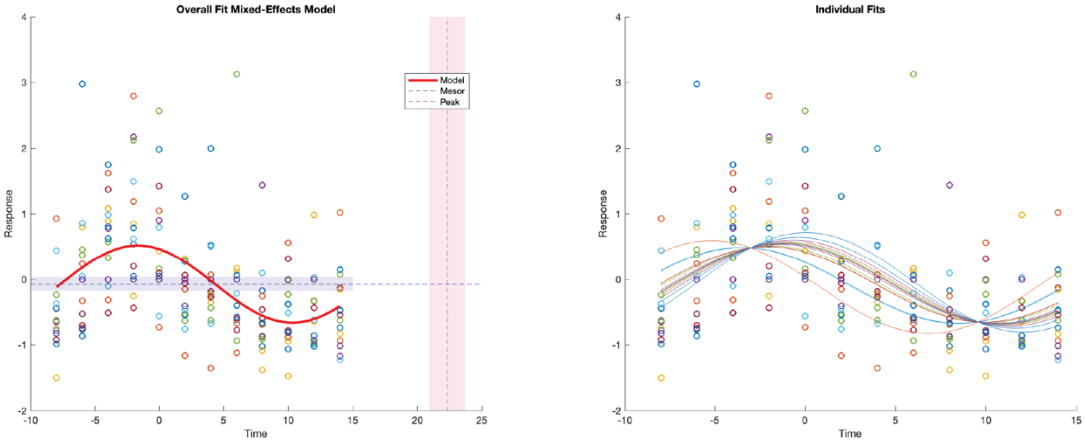

Deoxycholic acid

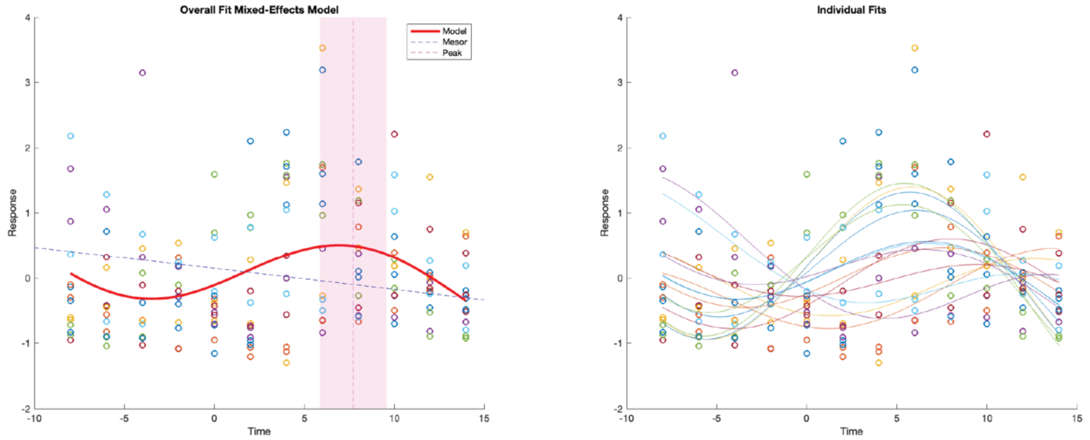

Glycodeoxycholic acid

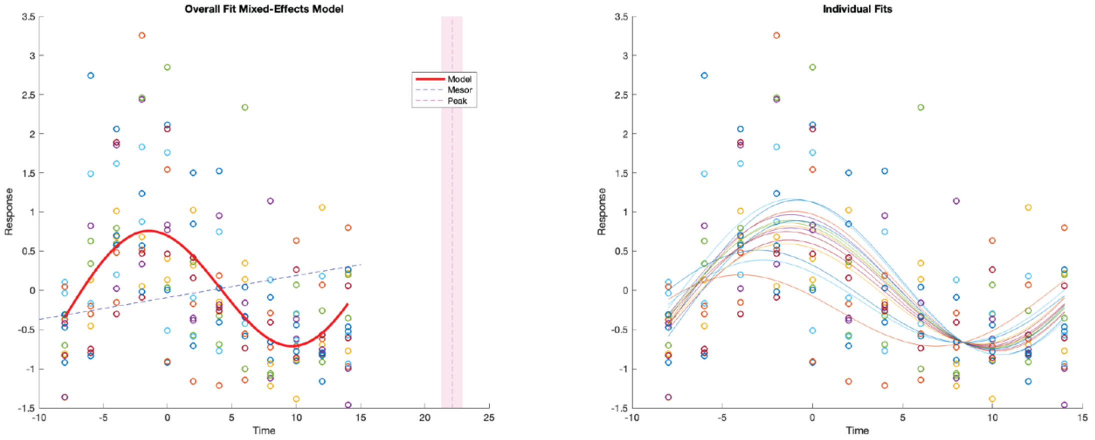

Taurodeoxycholic acid

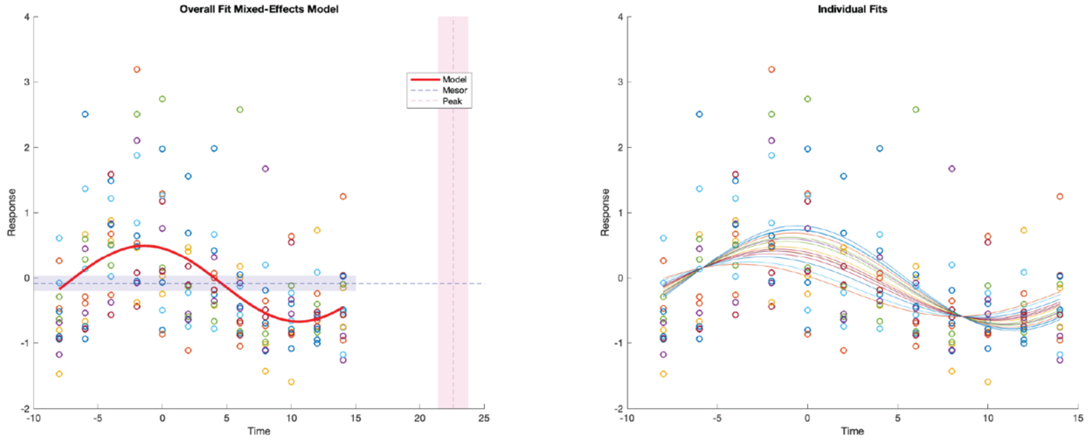

Lithocholic acid

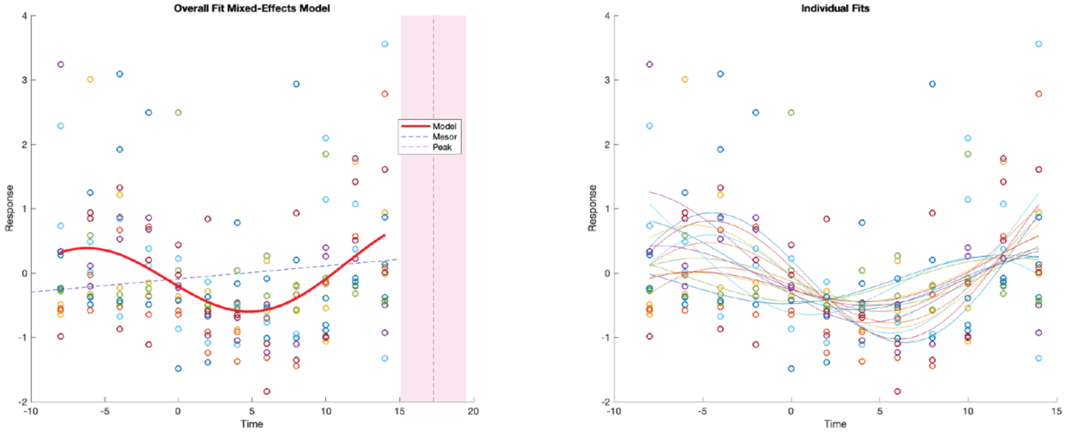

Glycolithocholic acid

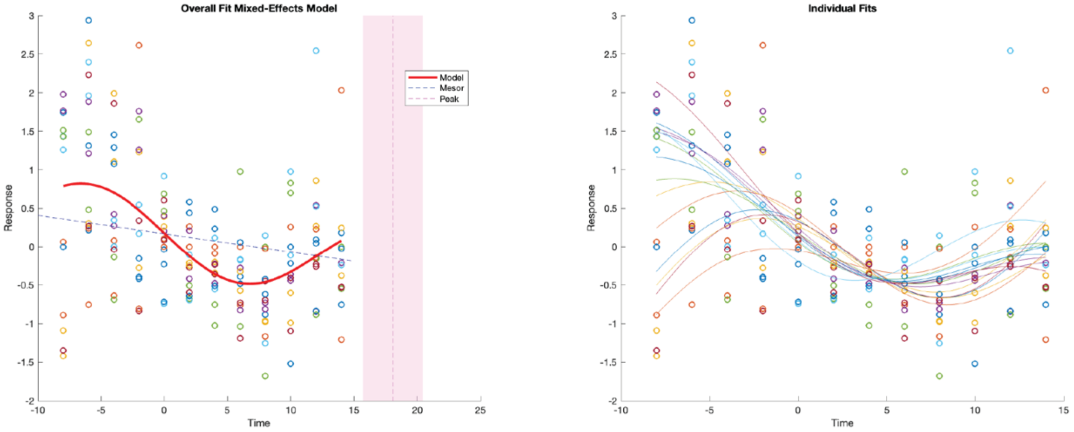

Taurolithocholic acid

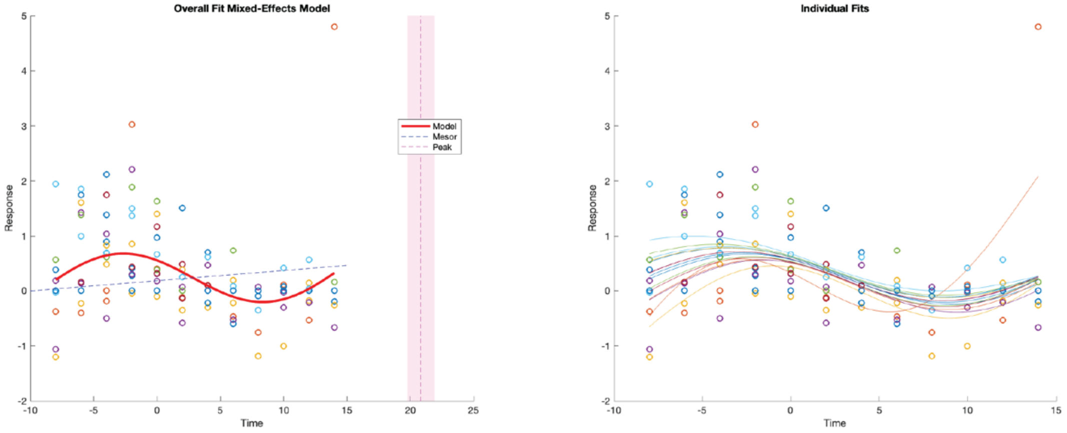

Ursodeoxycholic acid

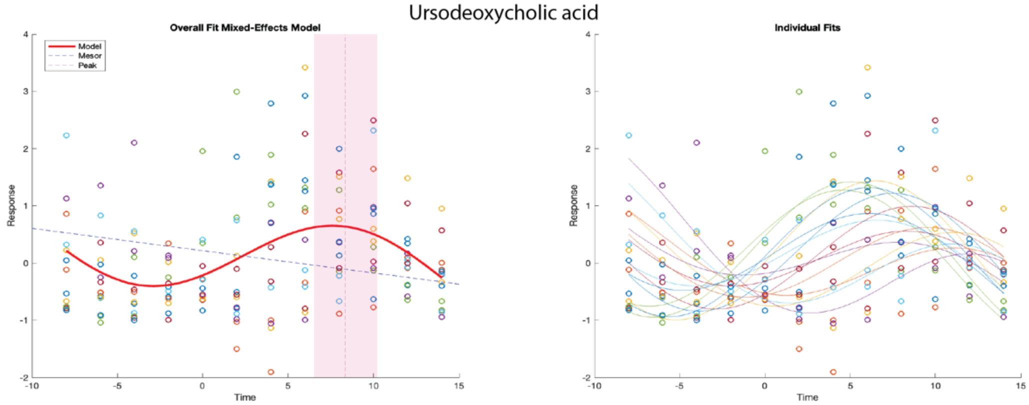

Glycoursodeoxycholic acid

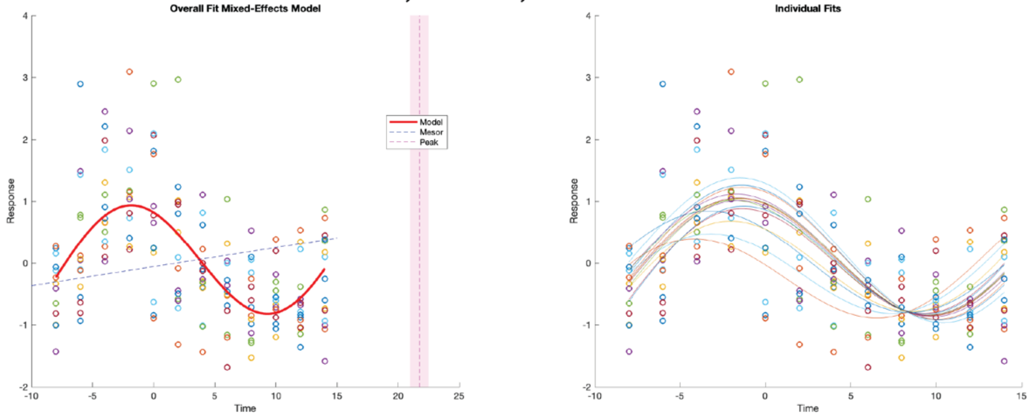

Hyocholic acid

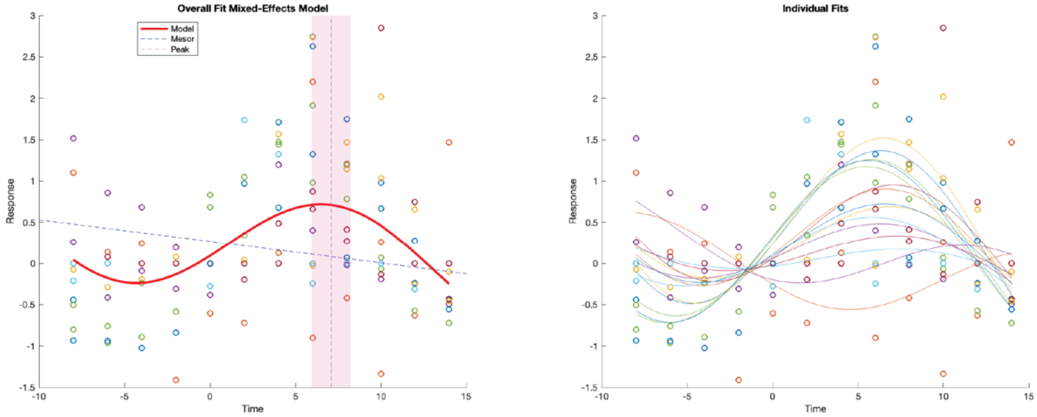

Glycohyocholic acid

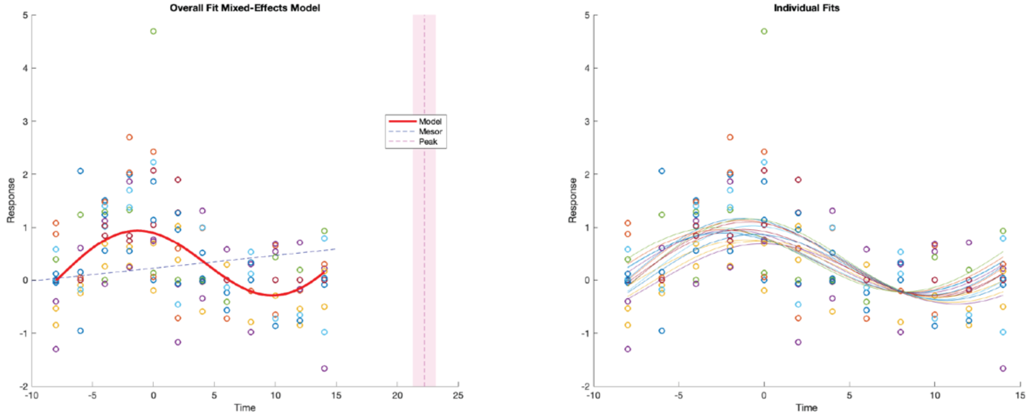

Supplementary Figure S2. Relative abundance of individual plasma bile acids within each individual in the entrained study (entrained+sleep and entrained+24h wakefulness).

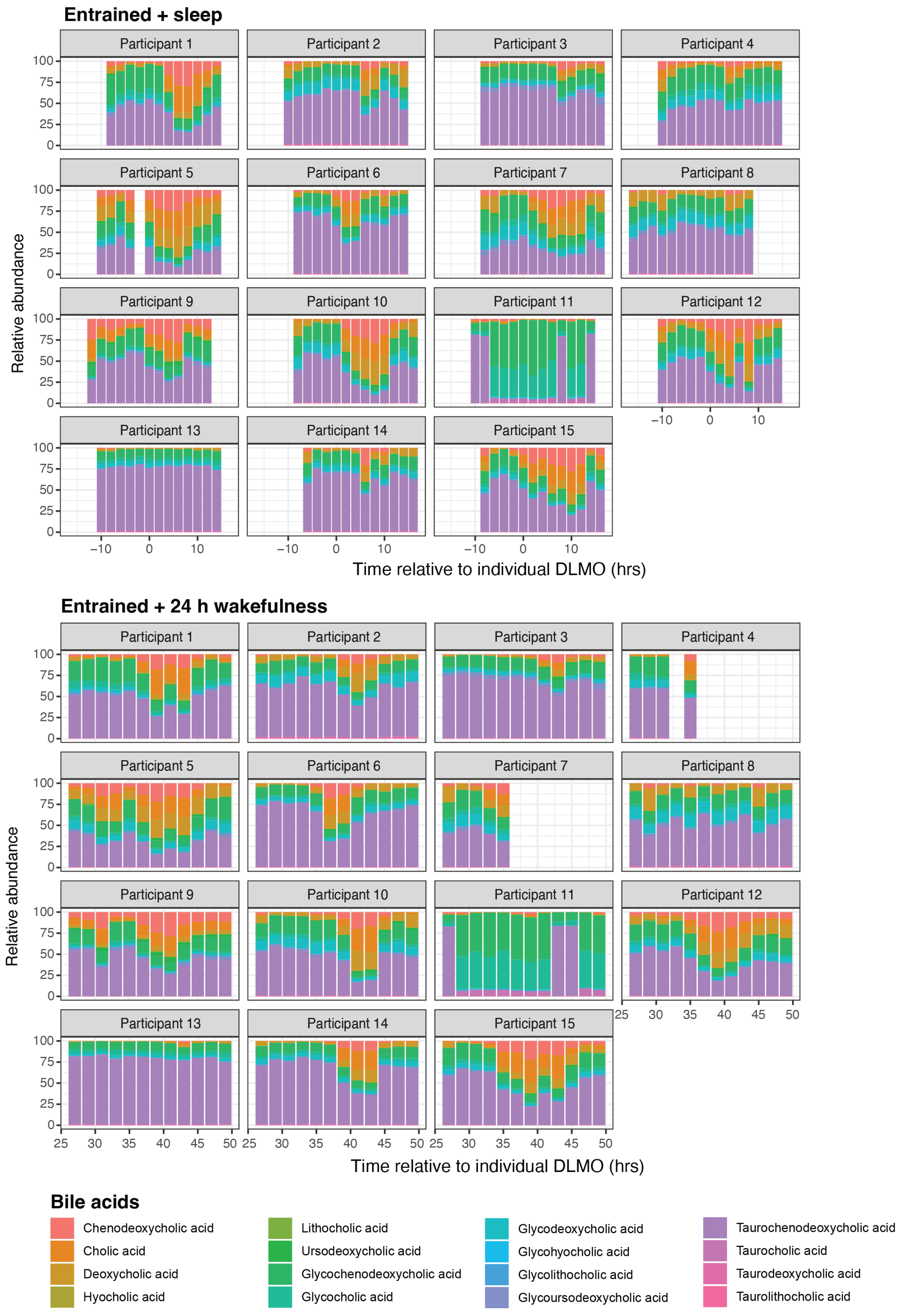

Supplementary Figure S3: Bile acid rhythms (entrained + entrained with sleep deprivation; n = 15).

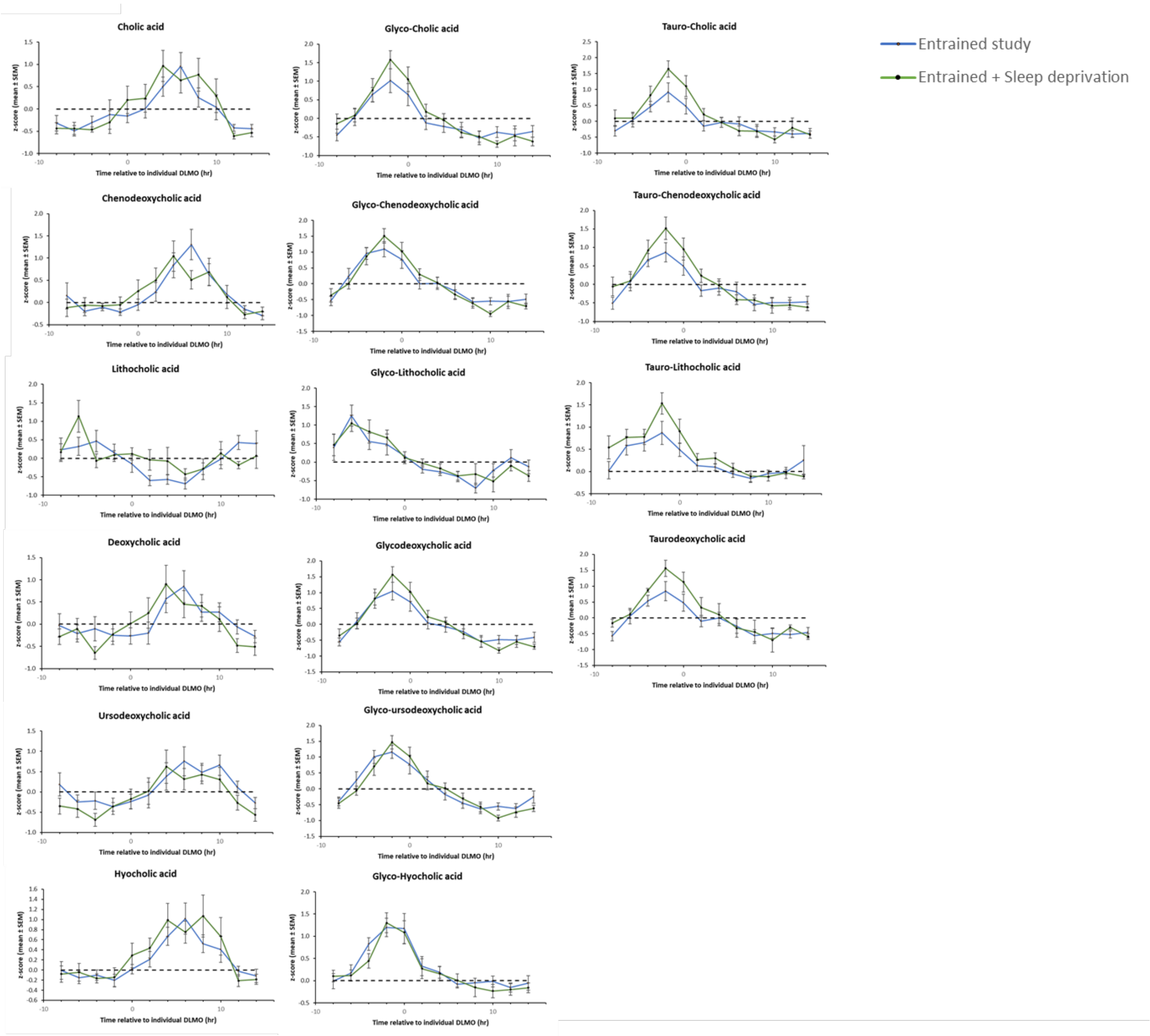

Supplementary Figure S4: Constant routine study protocol.

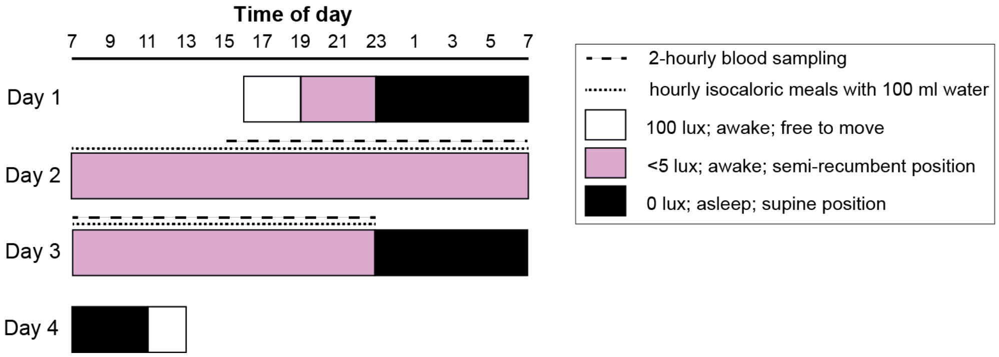

Supplement: Supplementary file 1 — Supplementary Information [file 41467_2024_53673_MOESM1_ESM.pdf]
