# Supplementary material for: Exposing 24-hour cycles in bile acids of male humans
Source: Nat Commun. 2024 Nov 19;15:10014. doi: 10.1038/s41467-024-53673-9 (PMC11576969; doi:10.1038/s41467-024-53673-9)
Supplement: Supplementary file 3 — Description of Additional Supplementary Files [file 41467_2024_53673_MOESM3_ESM.pdf]

Description of Additional Supplementary Files

Supplementary Data 1: Study participant characteristics. Data shown for individuals from the entrained and constant routine studies.

Supplementary Data 2: Macronutrient composition for meals in the entrained protocol (sleep and 24h wakefulness).

Supplementary Data 3: Acrophase time of each circulating bile acid and the mean minimum and maximum concentrations of each bile acid in the entrained protocol. Values are mean acrophase (peak) time in decimal hours relative to DLMO. The minimum and maximum concentration of each bile acid was calculated across the 24 h period for each individual and the means of these values across all individuals are presented (n = 15).

Supplementary Data 4: Significant correlations between circulating bile acids and metabolites and their phase difference. Phase difference reported in hours.

Supplementary Data 5: Acrophase time of each circulating bile acid and the mean minimum and maximum concentrations of each bile acid during 24 hours of wakefulness. Values are mean acrophase (peak) time in decimal hours relative to DLMO. The minimum and maximum concentration of each bile acid was calculated across the 24 h period for each individual and the means of these values across all individuals are presented (n = 15).

Supplementary Data 6: Impact of 24h wakefulness on bile acid-metabolite relationships. Average shift indicates the mean shift in the relationship between the specified metabolite and all bile acids following 24 h wakefulness. Similarly, bile acid average shift indicates the mean shift in the specified bile acid against all metabolites following 24 wakefulness. Table displays only the upper quartile of these shifts.
